# Biogas impurities: environmental and health implications, removal technologies and future perspectives

**DOI:** 10.1016/j.heliyon.2022.e10929

**Published:** 2022-10-06

**Authors:** Adhena Ayaliew Werkneh

**Affiliations:** Department of Environmental Health, School of Public Health, College of Health Sciences, Mekelle University, P.O. Box 1871, Mekelle, Ethiopia

**Keywords:** Biogas, Biogas impurities, Biogas and health, Biogas upgrading and cleaning, Biomethane

## Abstract

Biogas is a promising bioenergy alternative to be recovered from waste/wastewater in the context of environmental sustainability and circular economy. However, raw biogas contains various secondary impurities such as carbon dioxide, hydrogen sulphide, siloxanes, nitrogen oxides (NOx), ammonia, and halogens. Depending on the emission rate of these biogas impurities, the importance of biogas is being hampered for its environmental, health and the detrimental effects possess by the impurities towards the downstream of the biogas users. Biogas impurities can cause different public health concerns (like pulmonary paralysis, asthma, respiratory diseases and deaths) and environmental impacts (such as global warming, climate change and their indirect impacts like drought, flooding, malnutrition and other disasters). The absence/inconsistent emission standards among countries, agencies, and other stakeholders is the other challenge that they possess during monitoring and controlling of these impurities. Different commercially available and emerging technologies are available for separating carbon dioxide (via biogas upgrading) and removing other biogas impurities. Technologies such as pressure swing adsorption, membrane separation, absorption-based techniques (water, chemical and physical organic solvents), cryogenic separation, and other emerging biotechnological platforms (like photobioreactor and biocatalysis) have been adopted in removing the impurities. This paper reviewed the main commercially available and new technologies and their performance in removing carbon dioxide (the main constituent of biogas) and other biogas impurities. Besides, the environmental and public health implications of biogas and future research perspectives are also highlighted.

## 1 Introduction

Development of clean energy from sustainable sources (waste sector) is a global interest of our time to mitigate the issue of environmental degradation and climate change (Carranza-Abaid et al., 2021; Kohlheb et al., 2021). This causes a paradigm shift in the circular economy perspective of organic waste and wastewater management (Atelge et al., 2021; Paglini et al., 2022). In this approach, the conventional thinking of waste as a disposable material is no-longer appropriate, but the organic waste/wastewater is a resource for bioenergy production through the process of anaerobic digestion (Nguyen et al., 2020). The implementation of anaerobic digestion is an encouraging success and has become an impetus for its simultaneous applications in waste management and biogas production (Wickham et al., 2018; Paolini et al., 2018b; Kohlheb et al., 2021).

Biogas is a biofuel produced by a huge number of anaerobic microbial species that possess the ability to biodegrade organic matter under controlled moisture, temperature, and pH to produce a higher energy value fuel under anaerobic conditions (Grande et al., 2011; Chen et al., 2015; López et al., 2012). Recent research has approved that the development and usage of biogas have a vital contribution to reduce global warming (Tan et al., 2017; Ardolino and Arena, 2019; Wasajja et al., 2020; Paglini et al., 2022). Biogas is used to generate electricity in gas turbines and is considered an environmentally friendly and clean energy source when compared to non-renewable sources (fossil fuel from coal) (Hoyer et al., 2016). However, biogas utilization has limited due to its composition, which depends on the type and origin of feedstock, treatment processes (digestion process), and the process parameters (Table 1) (López et al., 2012; Kaparaju, 2013). This implies that the potential calorific value of methane in the raw biogas could be different (Mattioli et al., 2017; Wickham et al., 2018). This raw biogas mainly contains 40–75% methane and 15–60% carbon dioxide (Atelge et al., 2021; Macor and Benato, 2021; Carranza-Abaid et al., 2021; Dannesboe et al., 2021). However, the rest are secondary impurities such as 0–15% nitrogen (N_2_), <0.6% carbon monoxide (CO), 0–10000 ppm_v_ hydrogen sulphide (H_2_S), 0–3% oxygen (O_2_), 0–100 ppm_v_ ammonia (NH_3_), 0–41 mg Si/m^3^ siloxanes, 0–200 mg/m^3^ hydrocarbons, 1–5% water (H_2_O) and other particulates (Persson et al., 2007; Bauer et al., 2013; Ryckebosch et al., 2011; Awe et al., 2017; Mattioli et al., 2017, Wickham et al., 2018). Several countries have their own biogas quality requirements for injection into the natural gas grid or/and vehicle fuel utilization (Allegue and Hinge, 2012; Macor and Benato, 2021). The energy content of biogas is determined by methane composition (i.e. the higher methane concentration leads to a higher calorific energy value) (Chen et al., 2015; Kohlheb et al., 2021) (Table 1). For example, according to the Wobbe index, the calorific value of biogas with 70% and 100% methane and biomethane content yields 21.5 and 35.8 MJ/Nm^3^, respectively (Nguyen et al., 2020). However, the presence of a high content of carbon dioxide (CO_2_) and other trace gases reduces the economic value, its heat content and limits beneficial applications of biogas (Xu et al., 2017; Atelge et al., 2021).

**Table 1:** Main composition of raw biogas from different waste sources with their calorific value and corresponding effects (adapted from Kaparaju, 2013; Awe et al., 2017).

Parameter	Farm-scale AD plant	Centralized AD plant	Landfill gas	Sewage treatment	Natural gas (Holland)	Biomethane
Methane (% vol.)	55–60	60–70	30–65	60–65	81–89	>97
Carbon dioxide (% vol.)	35–40	30–40	25–45	35–40	0.67–1	<2
Hydrogen sulphide (ppm)	25–30	0–2000	30–500	<0.5–6800	0–2.9	3.5 ± 1.5∗
Water vapor (% vol.)	–	1–5	1–5	–	–	–
Hydrocarbons (% vol.)	0	0	0	0	3.5–9.4	–
Hydrogen (% vol.)	0	0	0–3	0	–	<0.5
Nitrogen (% vol.)	<1–2	2–6	<1–17	<1–2	0.28–14	
Oxygen (% vol.)	<1	0.5–1.6	<1–3	<0.05–0.7	0	
Ammonia (ppm)	≈100	≈100	≈5	<1–7	0	0.25 ± 0.01∗
Halogens (as Cl^−^ in mg/m^3^)	<0.01	<0.25	0.3–225	0–2	–	
Siloxanes (mg/m^3^)	<0.03–<0.2	<0.08–<0.5	<0.3–36	<1–400	–	
Wobbe index (MJ/m^3^)	24–33	24–33	20–25	25–30	44–55	
Lower heating value, (MJ/Nm^3^)	23	23	16	22	31–40	

Note: ∗ – mg/m^3^.

Accordingly, if biogas is not treated for the removal of its impurities (prior to its high value applications), the heat value of biogas will drop (due to its lower calorific value during combustion by reducing the biogas methane content) and these impurities will cause environmental impacts (Paolini et al., 2018b) and health concerns (Macor and Benato, 2020a, 2020b, 2021). Different public health concerns (like pulmonary paralysis, asthma, respiratory diseases, the spread of communicable diseases and deaths) and various environmental impacts (such as global warming, climate change and their indirect impacts like drought, flooding, malnutrition, and other disasters) have been reported (Macor and Benato, 2020a, 2020b, 2021) (Table 2). According to the Health Impact Assessment (HIA) explained by Macor and Benato (2021), the biogas impurities, NOx, SOx, volatile organic compounds (VOC) and CO contributes to 91, 6.5, 1.4 and 0.7%, respectively to damage human health. With respect to effects into the biogas upgrading process, it can lead to the failure of process functions of pipelines, power equipment, connections, and nozzles, triggering the process equipment to corrode (Biernat and Samson-Bręk, 2011), as they tend to build-up or accumulate. A complete removal of carbon dioxide and other impurities is essential to enhance the biogas quality into biomethane (Nguyen et al., 2020) and to withdraw the revealed impacts. The need for biogas pretreatment is not only to address the aforementioned issues (Macor and Benato, 2020a, 2021), but also to increase its calorific value for use in high-value applications by converting it to biomethane (a quality equivalent to natural gas) (López et al., 2012). Therefore, there is a need to apply sustainable technologies based on economic, environmental, and health implications of biogas.

**Table 2:** Effects of some common biogas impurities to human health, environment and process equipment.

Biogas impurities	Threshold limit to health and the env't.	Effects on human health and the environment	Effects to process equipment	References
Hydrogen sulphide	^1^Odor: < 0.014 mg/m^3^ Few days of eye irritation and respiration irritation: < 1 ppm.^2^	^2,5,9^Hazardous to environment, forming SO_2_ and SO_3_ and acid rain (H_2_SO_4_) Bad smell^3,^ eye irritation and unhealthy^1,2,4^ H_2_S is a potent nerve poison^9^	Highly corrosive,^6,7^ poisoning to the catalytic converter^8^ Toxic to the PSA adsorbents^9^	^1^Drimal et al. (2010),^2^Rubright et al. (2017),^3^Axelsson et al. (2012),^4^Roth (2004), ^5^Lim et al. (2016), ^6^Guidotti (1994), ^7^Tang et al. (2010), ^8^Awe et al. (2017), Paolini et al. (2018b), ^9^Atelge et al. (2021),
Siloxanes	-	No environmental effect^1^, only “responsible for fouling in the post-combustion emissions control catalytic system”.^2^	Formation of “glassy micro-crystalline silica” that decreases life span of equipment.^2,3^	^1^Graiver et al. (2003),^2^Soreanu et al. (2011),^3^Arnold (2009)
Ammonia	Max. emission rate: <50 ppm_v_.^1^ During combustion, NH_3_ converts into NOx: <10%.^2^	Formation and emission of NOx after combusion.^3^ Toxic to the anaerobic bacteria^6^ Health problems, toxic and bad smell^6^	Corrosion, anti-knock properties to engines^5^, NH_3_ is less corrosive than H_2_S.^4^	^1^Mojtahedi and Abbasian (1995), ^2^Wendt and Sternling (1974), ^3^Ranalli (2007), ^4^Francis (1985), ^5^Awe et al. (2017), ^6^Atelge et al. (2021)
Nitrogen	-	No harmful environment effect.^1^	Reducing the calorific value, corrosion and anti-knock properties^2^	^1^Wasajja et al. (2020);^8^Awe et al. (2017)
Water vapour	-	Water vapour forms acids with CO_2_ and H_2_S.^2^	^1^Corrosion due to the reactions with NH_3_	^1^Awe et al. (2017)
Particulate matter	<20 μg/m^3^ for 24 h and for particle size of <2.5 μm < 2.5 μm.^1^	-	- Plugs the gas system and pores of the adsorbent.^2^	^1^Buysman (2015);^2^Williams et al. (2014)

The biogas has to be improved into biomethane via different raw-biogas purification methods aimed at carbon dioxide removal and cleaning of other impurities prior to utilization. In this way, pressure swing adsorption (PSA) and water scrubbers are the most commonly used technologies in biogas upgrading (Kohlheb et al., 2021). During the earliest biogas upgrading developments, the PSA and water scrubbing techniques are considered as the most dominant technologies in small scale operations (Sun et al., 2015; Kohlheb et al., 2021). Currently, a wider range of physico-chemical biogas upgrading technologies such as membrane and cryogenic separations, chemical and physical organic scrubbers (Biernat and Samson-Bręk, 2011; López et al., 2012; Hoyer et al., 2016; Xu et al., 2017; Nguyen et al., 2020) and other emerging biological technologies (photobioreactor) have been adopted (Mann et al., 2009; Rodero et al., 2019; Nguyen et al., 2020; Debowski et al., 2021; Toledo-Cervantes et al., 2022). Selection of the best appropriate technologies among the available processes are performed through a variety of conditions such as required degree of purity, composition of the raw biogas to be treated, scale of operation and operational cost (Riboldi and Bolland, 2015; Baena-Moreno et al., 2019), where not a single technique is being more/less essentially than the other (Brunetti et al., 2010; Vrbová and Ciahotný, 2017; Maurya et al., 2019; Carranza-Abaid et al., 2021). This paper reviews the existing and emerging technologies and their performance in removing carbon dioxide (the main constituent of biogas) and other biogas impurities. In addition, the environmental and public health implications of biogas and future research perspectives are also discussed.

## 2 Biogas impurities

Biogas, which is produced through the anaerobic digestion of biodegradable waste/wastewater is typically composed of mainly methane and carbon dioxide and a variety of impurities such as H2S, water vapour, siloxanes, NH_3_ and VOC (Wasajja et al., 2020), where their composition differs with the source of their feedstock (Surendra et al., 2014; Saadabadi et al., 2019) and the type of biomass fed into the biogas producing digester (Wasajja et al., 2020). These biogas impurities have detrimental effect in the biogas conversion devices and possess a harmful consequence to human health and the environment as emissions when their presence are above their threshold limits (Papurello et al., 2016; Paglini et al., 2022) (Table 2). This section provides the description of the main biogas impurities and sources during the biogas production. Carbon dioxide (CO_2_) is the main component of biogas next to methane, which produced in some steps during the biogas production process. CO_2_ can be utilized by the methanogenic microorganisms to be act as an electron acceptor as, where its composition in the raw biogas is influenced by various operating conditions such as pressure, temperature and digester liquid content (Atelge et al., 2021). The formation of carbonic acid when water is mixed with CO_2_, causes to damage the process equipment Deng et al. (2020); Atelge et al. (2021).

Hydrogen sulphide, is an odorous gas and the most plentiful constituent of biogas, which is toxic for both to human health and the environment quality (Paglini et al., 2022), where it's corrosively characteristics lead to damage the biogas-conversion devices and end-users (harms the internal composition engines and the pipelines of the gas transport (Lanzini et al., 2017). The main source of H_2_S is from organically bounded sulfur compounds from proteins and SO_4_^2−^ through sulfate reducing bacteria (SRB) (Du and Parker, 2012; Van Loosdrecht et al., 2016) is while with the contribution of biomass type, the concentration of H_2_S could reach up to several thousands of ppm_v_ (Table 1) (Lanzini et al., 2017; Wasajja et al., 2020; Paglini et al., 2022). When sulphate is present during the anaerobic digestion, H_2_S is always produced by SRB (Rasi et al., 2007; Papadias et al., 2012; Wasajja et al., 2020). Hydrogen sulphide could be a source of sulfur oxides formation, NOx (SO_2_ and SO_3_) through oxidation in the combustion engines and boilers, which are released into the atmosphere as emissions along with exhaust gas (Niakolas, 2014; Paglini et al., 2022). The toxic and irritative nature of both SO_2_ and SO_3_ towards mucous membrane affects human breathing system (Munawer, 2018; Khan et al., 2019; Paglini et al., 2022).

Siloxanes are highly water souluble and lead to be the second type of biogas impurities having a significant concern (De Arespacochaga et al., 2015; Wasajja et al., 2020) during its applications. Siloxanes are semi-volatile organic compounds which contains silicon and used in a number of cosmetics industry such as deodorants, detergents, food additives and soap (McBean, 2008; De Arespacochaga et al., 2015; Paglini et al., 2022) and widely spread within the environmental components (Arnold and Kajolinna, 2010; Wasajja et al., 2020). This makes that siloxanes are abundant in the sludge of the wastewater treatment plants at the same time contributed as the main constituent of biogas (Lanzini et al., 2017). At higher temperature of the anaerobic digester for biogas production, the volatilze nature of siloxanes could be entered as a biogas constituents (Papadias et al., 2012; Madi et al., 2015; Lanzini et al., 2017; Wasajja et al., 2020; Papadias et al., 2021; Paglini et al., 2022). The composition of siloxanes in the landfill gas and WWTs are expected to contain a maximum of 4–9 ppm and high as 41 ppm, respectively (Arnold and Kajolinna, 2010). However, the biogas from farm digesters also contains the least amount of siloxanes, while no information could found from small scale biogas digesters (Wasajja et al., 2020).

Volatile organic compounds (VOC) are other constituents of biogas, which includes alkanes, alcohols, halogens and aromatic compounds, which are found at various concentration levels depending on the conditions of anaerobic digestion and type of biomass ((Papadias et al., 2012; Paglini et al., 2022). The downstream equipment and pipelines are also affected by halogens, mainly due to the corrosive products formed by the reaction of halogens (like chlorine) with Ni-based catalysts (on the anode side) “followed by the sublimation of the reaction product” (for example, gas phase NiCl_2_ for Cl_2_ poisoning) **(**Papadias et al., 2012; Sitthikhankaew et al., 2014). However, the composition of chlorine in the raw biogas is too low, thereby no further treatment is required for its removal (Bona et al., 2020; Rout et al., 2021; Paglini et al., 2022).

## 3 Effects of impurities in the biogas upgrading processes

The presence of a high volume of carbon dioxide in the biogas not only reduces the calorific value but also makes the biogas not economically viable for transportation and compression during offsite utilization. The biogas impurities can be detrimental to the downstream utilization processes, where hydrogen sulphide is corrosive to the co-generators, compressors, biogas storage facilities, and pipelines. Besides, the combustion of hydrogen sulphide produces major air pollutants such as sulfur dioxide. However, the European biomethane standard sets the required threshold concentration limit of hydrogen sulphide to be less than 1 pmv for injection gas grid and transport fuel (Nguyen et al., 2020). Raw biogas is commonly saturated with water once leaving the digester, and it causes a problem as it may condensate in the gas pipelines when passing from higher to lower pressures, causing corrosion and clogging together with sulphur compounds (like hydrogen sulphide), affects and destabilizes the structure of the adsorbent materials during the purification process via activated carbon (Petersson and Wellinger, 2009a, Petersson and Wellinger, 2009b; López et al., 2012). Besides, water vapor causes a trick during vehicle fuel or grid injections later in the biomethane applications. Accordingly, the pipeline quality standards need 100 mg/m^3^ of water content, while “compressed natural gas vehicle fuel standards” also desire 10 °C dew points (Ryckebosch et al., 2011).

The permissible content of water vapor is below 10 mg/Nm^3^ for injection into the gas grid (Nguyen et al., 2020). In addition to these impurities, the presence of siloxanes in biogas can lead to the formation of siloxane dioxide particles, having an abrasive and adhesive property to metal surfaces, causing excessive tear and wear of co-generator engines, while its maximum permissible concentration limit in natural gas is 5 mg (Si)/Nm^3^ (Nguyen et al., 2020). The halogenated hydrocarbons, mainly chlorine, bromine, and fluorine based compounds, are frequently found in the biogas from landfills, while they are rarely present in the biogas from the digestion of organic wastes or sewage sludge. Halogens are corrosive and, during the combustion of biogas, can be the precursors to the formation of dioxins and furans (López et al., 2012), which pose public health risks.

## 4 Environmental and health implications of biogas

Half of the population, and specifically in developing countries, up to 95% of the population, relies on the energy supply based on solid fuels (biomass fuels) to meet their energy requirements, such as animal dung, wood, coal, and agricultural residues, while facing indoor air pollution that causes millions of deaths every year (Abadi et al., 2016). The World Health Organization lists “indoor air pollution from primitive households’ cooking fires as the leading environmental cause of death in the world, contributing to nearly 4.3 million deaths annually, or about as many as tuberculosis and malaria combined together” (Abadi et al., 2016). Cooking with biomass fuel is a major contributor to increased carbon dioxide emissions, resulting in climate change and a variety of public health concerns. However, currently, biogas technology has been developing rapidly to substitute the drawbacks of wood based solid fuels in terms of health and environmental implications.

Hence, biogas protects the environment (water, air, and soil), is a profitable renewable energy resource, and is responsible as a safe waste management solution, having a net positive impact in terms of cost and environmental friendliness compared with the non-renewable alternatives (Abadi et al., 2016). Due to the convenience and adaptability of use, biogas can be an opportunity for both developing and developed countries in terms of reduction of fossil fuel dependent energy supply, mitigating the effect of climate change and reduction of greenhouse gas emissions. Besides, biogas is an attractive alternative energy supply pathway for those countries that have a strong dependency on fossil fuel energy supply (Macor and Benato, 2020a).

However, the increasing emission of carbon dioxide into the atmosphere is the main contributor to environmental crises (Debowski et al., 2021). Impurities in biogas have several health implications (Macor and Benato, 2020a, 2020b, 2021) as well as environmental consequences (Paolini et al., 2018b). The biogas impurities, for example, carbon dioxide and NOx, have different environmental impacts such as climate change that results in malnutrition, flooding, the spread of communicable diseases and other disasters. With respect to the public health point of view, for example, hydrogen sulphide, which is among the main contaminants of biogas, is characterized as heavier than air, highly toxic and flammable gas, while upon inhalation, it reacts with the biological enzymes within the blood stream and results in inhibiting cellular respiration to cause sudden collapse, pulmonary paralysis, and death (López et al., 2012). The odor threshold limit of hydrogen sulphide is about 0.00047 ppmv (Aroca et al., 2007), whereas at higher concentrations of 200–300 ppm, the hydrogen sulphide may cause respiratory arrest that leads to unconsciousness (Syed et al., 2006). Due to the corrosive and toxic nature of hydrogen sulphide, sulfur dioxide emissions are caused by biogas use in combustion (López et al., 2012). As it was described by Macor and Benato (2020a), biogas is on average 10 times more toxic than natural gas in terms of dioxins and furans toxicity, and exhausts three times more NOx emissions than the natural gas standard. SOx emissions contributed about 6% of the imposed biogas human health toxicity.

As a general overview, human health damage from biogas impurities has been imposed through regulated and unregulated emissions, where "the regulated emissions are substances that have regulatory limits for their maximum levels, while the unregulated emissions (which have higher toxicity impacts) are compounds without fixed regulatory limits" (Macor and Benato, 2020b). Those of the biogas impurities considered as regulated emissions are SOx, NOx, HCl, CO, VOCs, particulate matters, while the polycyclic aromatic hydrocarbons, furans, dioxins, and aldehydes are considered as unregulated emissions. Furthermore, SOx, NOx, and H_2_S are responsible for human health damage (Macor and Benato, 2020a, 2020b, 2021). The main challenges of the biogas impurities to human health are mainly due to the absence of a widely adopted and common emissions standard (e.g. at EU level) for both regulated and unregulated emissions. This is a point of concern for the stakeholders, as each country (mostly in developed nations) independently defines the impurities that need to be "tracked and their maximum levels", which is considered the most challenging (Macor and Benato, 2020a, 2020b).

## 5 Removal technologies for carbon dioxide from biogas

The basic principle of biogas upgrading is to concentrate methane by separating carbon dioxide and removing other gaseous impurities such as hydrogen sulphide, water, nitrogen, hydrogen, VOC, and oxygen from the inlet raw biogas (Hoyer et al., 2016). There are different kinds of carbon dioxide separation technologies, which are classified according to the physico-chemical mechanisms utilized during their separation (Hoyer et al., 2016), while their maturity varies widely. It is advantageous that some technologies result in the simultaneous removal of both carbon dioxide and other impurities, while others require a pretreatment unit to remove biogas impurities. For example, PSA and water scrubbing (mainly at higher pH) remove both carbon dioxide and hydrogen sulphide simultaneously, while in chemical scrubbing and membrane separation techniques, a pre-treatment step is required for the removal of hydrogen sulphide using amines prior to carbon dioxide separation, and it is essential to avoid membrane poisoning (Allegue and Hinge, 2012; Nguyen et al., 2020). Then, the pre-treated biogas can be further processed to remove carbon dioxide to upgrade the biogas to biomethane level. In a multi-staged process, these technologies involve the following applications (Allegue and Hinge, 2012): i) An upgrading process with inert gases, mainly carbon dioxide, is captured to concentrate methane in order to meet the Wobbe index specifications, ii) During the cleaning process, trace constituents harmful to the natural gas grid end-users are removed. Various carbon dioxide separation technologies are available on the market, which include pressure swing adsorption, membrane separation, and absorption/scrubbing (i.e. water, chemical, and physical organic solvent) based absorption techniques, and other emerging technologies such as cryogenic separation and other emerging biological techniques (like photobioreactor) (Nguyen et al., 2020). This section describes how these technologies separate carbon dioxide from biogas to upgrade into biomethane and their performance in removing different impurities during the pre/during/post-treatments. Table 3 describes the comparisons among biogas upgrading technologies in removing carbon dioxide and other impurities.

**Table 3:** Comparisons between the biogas upgrading and cleaning technologies in removing carbon dioxide and other impurities (Adapted from Allegue and Hinge, 2012; Sutherland et al., 2019; Nguyen et al., 2020; Bose et al., 2020; Carranza-Abaid et al., 2021).

Methods	Opportunity	Limitations
Water scrubbers	- Its capacity is adjustable by varying the temperature and pressure - Several plants are operated worldwide (simple operation, cheap and simple method) - H_2_S (>300/500 ppmv) and ammonia co-removal (tolerance for impurities) - No additional heat required - Environmental friendly and low-cost solvent - Low operating cost - Achieved 95–99% biomethane purity	- High pressure (4–10 bars), methane loss (up to 5% by vol.), and energy consumption (is up to 0.2–0.5 kWh/Nm^3^ of biogas) - Pretreatment and drying of biomethane required - Clogging occurred due to the growth of bacteria - Medium content of biomethane produced, H_2_S (when >300/500 ppmv) damages the equipment - Higher water consumption (even within the regeneration process) - Loading and absorbent rate is too low (water is less selective), and a possibility to cause foaming
Solvent scrubbers	- Lesser footprint exists - Higher and effective absorption rate, and “higher loading per volume of solvent”	- Methane loss is up to 4% by vol., and heat is required for effective regeneration - Energy consumption is from 0.1-0.33 kWh/Nm^3^ of biogas) - Due to the environmental pollution of used solvents, additional post-treatment is required
Pressure swing adsorption	- No chemicals and no heat demand required - Cheap and compact technology, several plants are under operation, easy operation - Co-adsorption of N_2_ and O_2_ together with CO_2_ - Achieved 95–99% biomethane purity	- Medium amount of biomethane produced with medium/higher CH_4_ loss exist - “Extensive process control” and the use of valves often required - Pretreatment required for H_2_S and H_2_O, and attained 1–9% methane loss
Physical organic scrubbers	- Methane loss is low and coarse pretreatment step is required - Produces higher content of methane (energetic and more auspicious than water) - Co-removal of hydrogen sulphide, ammonia and other impurities - Achieved 95–99% biomethane purity	- Relatively expensive operation and investment cost - Difficulty in operation (can be reduced when the dilution of glycol with water exists) - Boiling required to avoid incomplete regeneration
Amine-based chemical absorption	- Higher efficiency of methane content - Process is carried out without pressure and no moving components required except lower - Low demand of electricity - Methane loss is very low - Dissolves more CO_2_ per unit of water (when compared with water) - Achieved >99% biomethane purity	- Quite an expensive investment required - High demand of heat required for regeneration - Causes corrosion, poisoning and decomposition of amines by oxygen occurred - Salts precipitation exists and foaming possibility - Pretreatment required for hydrogen sulphide removal - Lower working pressure (1 bar) and required heat - Process handling is complex and attained 1–2% methane loss
Membrane technology	- Easy operation and construction, and low maintenance - No moving components required except blower, no demand of heat or no chemical required, higher reliability - Small footprint and low weight - Modular configuration needed even at lower volume rates, Minor gas flows as treated without proportional increment of the cost - Acquired pure CO_2_ to be used in industrial applications - Gas-liquid provides cheap operation and investment cost - Achieved 95–99% biomethane purity - Low operational and moderate initial costs - Easy process handling when compared with others	- Medium methane contents produced, while multiple stages are needed to achieve higher methane purity - Medium to higher (10–15%) losses of methane (depending on membrane configuration) - Purity of methane is compromised with the amount of upgraded biogas - Requires petty operational experience for an improved membrane technology - Membrane cost is expensive, H_2_S removal step is required or pretreatment should be required - It is not suitable for biogas having many unknown contaminants (like from landfill) - Unsure membrane durability and low selectivity of membrane
Cryogenic separation	- Achieved higher methane content in the upgraded biogas - Lower methane loss exists - No chemicals added, and carbon dioxide produced as a byproduct - Requires lower extra cost of energy to “reach liquid biomethane” - Achieved up to 99% biomethane recovery potential	- Expensive operation and investment cost required - Further removal step required for siloxanes, hydrogen sulphide and other impurities - Technical skill is very demanding and the process handling is complex - The technology is still emerging - Pretreatment required and needs higher working pressure (40 bar) - Higher operational and initial costs. Methane loss is up to 1–2%
Photobioreactor	- Emerging Green technology (environmentally friendly), economically feasible - Applied in an integrated system with wastewater treatments (for removal of nutrients) - Sewage purification performance is too high - Higher carbon dioxide reduction and thereby increases in methane in the upgraded biogas.	- Higher methane loss because of its solubility within the microalgae culture and possess difficulty in harvesting the biomethane - Lack of tolerant microalgae species at higher carbon dioxide content; and oxygen introduction from the microalgae (photosynthesis) into the upgraded methane - Higher carbon dioxide content dissolves in water and thereby pH reduces to <6 to cause a detrimental effect to the growth of microalgae or disrupts the permeability of cell membrane and photosynthesis - Limiting solubility of carbon dioxide in the growth medium of microalgae and leads to lose up to 90% of raw biogas and this system is not yet validated at large scale operations

### 5.1 Pressure swing adsorption

**Figure 1:**
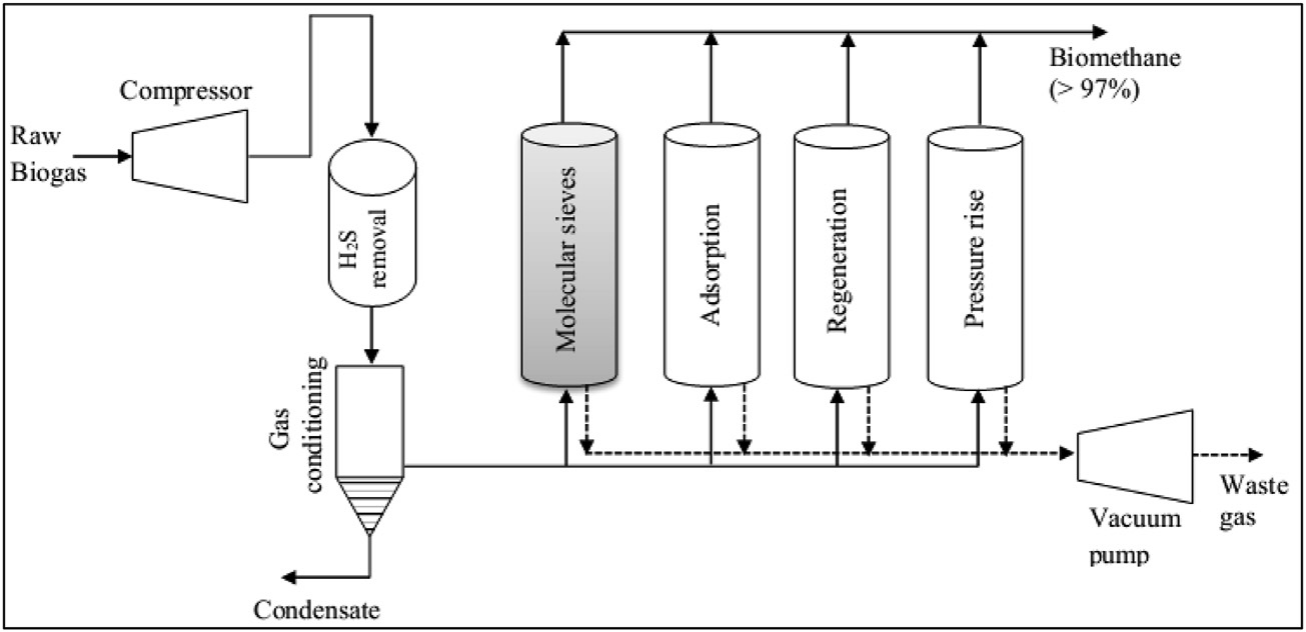
Schematics of pressure swing adsorption for biogas upgrading

This technology relies on the principle of adsorption that separates carbon dioxide from methane at different specific surfaces/pores of the adsorbents (Nguyen et al., 2020) based on their physical properties (Mel et al., 2016). The principle of this technology is that raw biogas is compressed at a raised pressure and then fed into an adsorption column that retains carbon dioxide while CH_4_ is not (Biernat and Samson-Bręk, 2011). This is because of the selective affinity of carbon dioxide on the surface of the adsorbent, which is done at different pressures (used for controlling the separation) (Hoyer et al., 2016). The pressure swing adsorption process utilizes preserved/temperature variations, where the adsorption of carbon dioxide is proportionally to low temperatures and high pressure (Ntiamoah et al., 2016). In this system, carbon dioxide is removed from biogas by adsorption on different surfaces of the adsorbents (such as activated carbon, calcium oxides, synthetic and natural zeolites, silica gels, hydrotalcites, and carbon molecular sieves) at increased pressure (Ntiamoah et al., 2016). Figure 1 shows the pressure swing adsorption (PSA) that illustrates several stages of unit operations in a series of vessels (i.e. filled with adsorption material, usually from 4-6) working on four different alternating cycles called the adsorption, de-pressuring, regeneration, and pressure build-up columns (López et al., 2012).

During the adsorption process, biogas enters from the bottom side into one of the adsorbers within the PSA vessel, and when passing through the vessel, nitrogen, oxygen, and carbon dioxide are adsorbed by the media, and the gas exists as biomethane (Mel et al., 2016). Biogas goes to another ready vessel, which has already been regenerated to achieve continuous operation before the adsorbent material is completely saturated (Allegue and Hinge, 2012). The choice of the adsorbent and the bed material that selectively adsorbs carbon dioxide from the gas stream is very significant for the function of the PSA unit. Research and development in this section is focused on: (i) optimizing the PSA for small scale applications; (ii) reducing the number of PSA units; and (iii) reducing energy consumption (Hoyer et al., 2016).

The PSA technique requires a pre-treatment step in order to remove hydrogen sulphide and water vapor present in the biogas inlet stream (López et al., 2012). When the column material is saturated with carbon dioxide, then pressure is released and carbon dioxide can be desorbed and led into an off-gas stream (Hoyer et al., 2016). Adding more columns is required as they will be opened and closed sequentially and to optimize more advanced flows between the columns. This is the way to increase both methane energy and carbon dioxide separation efficiencies, but has difficulty in technological acceptability and investment costs (Hoyer et al., 2016). In the PSA process, hydrogen sulphide removal in the treatment step is needed using activated carbon filtration followed by an increase in temperature between 60 and 90 °C to which the gas-phase from biogas can be easily removed) (Schulte-Schulze, 2005), otherwise it irreversibly binds to the adsorption media at elevated pressure and causes damage, generates toxic effects, and possesses bed deactivation in the PSA column (Patterson et al., 2011; Nguyen et al., 2020). Water vapor can also destroy the adsorbent structure, therefore, its removal in the pretreatment is required through condensation after the desulfurization (López et al., 2012). Besides, volatile organic compounds (VOC) and ammonia from the raw biogas can be removed in the PSA adsorption column after the compression stage. The concentrated biomethane exiting the PSA scheme is dry enough, with a dew point of −50 °C, to be used without further drying (Hoyer et al., 2016).

The regeneration of the adsorbed material is usually done in several steps: i) the pressure is reduced by linking the vessel with an already regenerated vessel, ii) the pressure is reduced to the atmospheric standard values, and iii) the vessel is completely evacuated using a vacuum pump (Petersson and Wellinger 2009; López et al., 2012). The typical adsorption and regeneration pressures in the PSA are in the range of 3–7 bar and 100–200 mbar, respectively, while its temperature range is 50–60 °C (Hullu et al., 2008). Accordingly, carbon dioxide adsorption takes place at an optimum pressure, while the adsorbent regeneration is done at a reduced pressure and by the subsequent application of light vacuum (Hoyer et al., 2016). After exploiting the adsorption capacity, the adsorbent material can be regenerated by direct heating of the gas to the boiling point of the adsorbent in the PSA column or by injecting hot air, nitrogen gas, or steam into the column (Biernat and Samson-Bręk, 2011; Ntiamoah et al., 2016). The decomposition of substances occurs by lowering the pressure in the system (since pressure is reduced gradually during the regeneration process). Then, the gas that is adsorbed is recycled back into the tank together with the raw biogas, because a certain recoverable amount of methane might be adsorbed together with carbon dioxide (Biernat and Samson-Bręk, 2011; Nguyen et al., 2020). The adsorbed material in the PSA column contains a waste stream including oxygen, nitrogen, and carbon dioxide along with a small fraction of methane that can be recirculated again into the inlet in order to recover methane, while the methane-free outlet gas-stream leaving the vessel contains mainly carbon dioxide (that can be released directly into the atmosphere or can be sent for further treatment). For example, the outlet gas-stream can be linked to a generator and, thereby, the emission of carbon dioxide into the atmosphere can be avoided (López et al., 2012).

The PSA has been operated as a common biogas upgrading plant for many years and achieved 96–98% biomethane quality with 1.5–2.5% methane loss and required 0.15–0.35 kWh/Nm^3^ overall energy of biogas (Allegue et al., 2012), while post-combustion of the exhaust gas is required to minimize the methane loss into the atmosphere (Nguyen et al., 2020). According to a study by Paolini et al. (2018a) a vacuum swing adsorption (VSA) (which is similar to the PSA but has the capacity for its stronger resistance to siloxane and hydrogen sulphide) onto synthetic zeolite has been demonstrated for the removal of carbon dioxide from biogas produced from sewage sludge, and achieved close to 99% removal efficiency of carbon dioxide. Augelletti et al. (2016) studied the performance of PSA technique for separating methane and carbon dioxide from biogas and achieved above 99% of biomethane recovery with the consumption of 1250 kJ/kg biomethane energy. As it was described by Schulte-Schulze (2005) and López et al. (2012), PSA is advantageous because it: i) produces 96% biomethane concentration and low levels of air emissions and solid waste, ii) simple and automated operations, iii) simultaneous co-removal of other impurities (silicon, halogenated compounds, and partial removal of nitrogen and oxygen), iv) no handling of chemicals or water, thereby no formation of wastewater streams. However, the PSA has some disadvantages (López et al., 2012): (i) waste gas and carbon dioxide emissions exist; (ii) a pretreatment unit for hydrogen sulphide removal and water vapor removal is required; and (iii) energy is required in the heat exchangers and compressors cycle of adsorption, pressure buildup, and regeneration.

### 5.2 Absorption/scrubbing techniques

#### 5.2.1 Water scrubbing techniques

**Figure 2:**
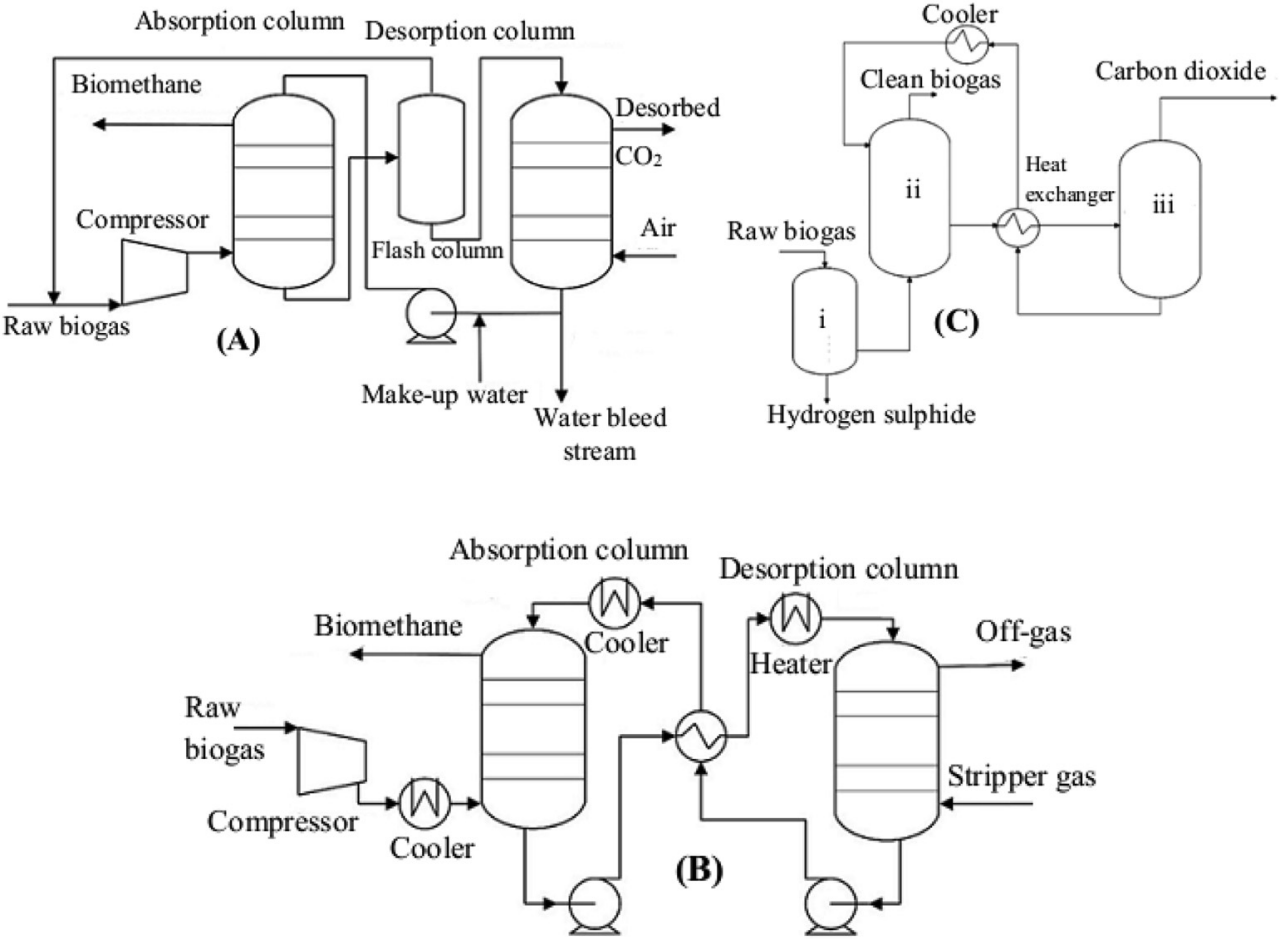
Schematics of the scrubbing techniques for biogas upgrading through carbon dioxide separation and removal of other impurities: (A) water scrubbers, (B) physical organic scrubbers, and (C) chemical scrubbers (i) - desulfurization, (ii) – absorption column, and (iii) – regeneration column (adapted from Hullu et al., 2008; Hoyer et al., 2016).

Water scrubbing (Figure 2) is the most popular biogas upgrading technique, and the plant equipment is commercially available from several suppliers in a broad range of capacities (Biernat and Samson-Bręk, 2011). This technique is a very simple process having lower energy consumption and high water consumption (200 m^3^/h for a gas flow of 1000 Nm^3^/h), with few rotating components at higher operational time (Sun et al., 2015), while the methane loss (3–5%) is the main drawback (Ryckebosch et al., 2011; Nguyen et al., 2020). In this system, water is used to separate carbon dioxide from biogas based on the principle that relies on the gaseous solubility difference between carbon dioxide and methane in a wash solution (water) and involves no chemical addition (Allegue and Hinge, 2012; Andriani et al., 2014). The pretreated biogas should be maintained at a 40 °C temperature and 6–10 bar pressure in the scrubbing column to yield the carbon dioxide solubility, which is approximately 26 times higher than methane (Hoyer et al., 2016; Nguyen et al., 2020). Methane is also dissolved in water, while its solubility is much lower than the biogas impurities (Allegue and Hinge, 2012), while the methane concentration in the gas-phase is going to be increased. In this process, raw biogas is cleaned from carbon dioxide, ammonia, and hydrogen sulphide, which are physically dissolved in water under increased and reduced pressure and temperature, respectively, in the absorption column to increase their solubility (Allegue and Hinge, 2012), and thereby the pollutants can be easily dissolved in the aqueous phase (López et al., 2012).

As shown in Figure 2A, the scrubbers involve two columns working in parallel in different stages (when the first column is filled, the second may be emptied). Biogas is compressed and injected via the bottom side of the column (Nguyen et al., 2020), while water is provided from the top side of the column, and the biogas impurities such as carbon dioxide and sulfur compounds are dissolved in it. In order to increase the contact surface between biogas and water, the column is filled with a packed material (Hullu et al., 2008; Andriani et al., 2014). When the cleaning process is accomplished, gas leaving the scrubber column, which has a much higher concentration of methane gas, has to be recovered and the carbon dioxide rich water is pumped into a stripping column for regeneration (removal of carbon dioxide from water) (Biernat and Samson-Bręk, 2011). The recovered methane is subjected to a drying step for biomethane formation (Angelidaki et al., 2018; Nguyen et al., 2020) and the water is recycled back to the adsorption column to be used for further carbon dioxide and hydrogen sulphide absorption. Water scrubbing technology is advantageous when applied at the WWT plants, because secondary and tertiary effluents can be used as a water source without the requirement of a regeneration process (Angelidaki et al., 2018; Nguyen et al., 2020).

There are two types of water absorption processes in the liquid stream; single pass absorption and regenerative absorption. In a single pass process (without regeneration), the washing water is used only once and the wastewater produced not only emits carbon dioxide into the atmosphere, but also hydrogen sulphide and methane. Having this justification, this technique is advantageous in that the inlet water is free of carbon dioxide and hydrogen sulphide. As it was noted by Allegue and Hinge (2012), this system is primarily used with biogas from wastewater treatment plants since they have access to the large supply of onsite water and wastewater treatment. The drawbacks of this technique is that it requires a huge amount of water, which produces a large amount of WW that has to be treated in the WWT plants, is not eco-friendly, and unprofitable in terms of cost. However, in the regenerative water scrubbing process, the washing water is regenerated through desorption after biogas is subjected to washing. When compared to the single pass scrubbing process, a significant reduction in the quantity of water used is achieved when the water can be recirculated in the system (Hullu et al., 2008; López et al., 2012), and in most cases, the regenerative absorption is preferable. More than 97% of biomethane can be produced (López et al., 2012). Furthermore, high concentrations of volatile sulfur compounds, chlorine and nitrogen can be removed easily under optimized process conditions.

#### 5.2.2 Organic physical scrubbing techniques

This technique (Figure 2B) is basically a robust technology, similar to the water scrubber in being able to handle various impurities, with the difference that in this scrubber, instead of water, organic solvents are used to absorb carbon dioxide and then ammonia, hydrogen sulphide and water can be separated (Allegue and Hinge, 2012). Carbon dioxide and hydrogen sulphide are more soluble in the organic solvent than in water, while the operational costs, wastewater treatment costs, solvent regeneration and pumping requirements will be reduced (Hoyer et al., 2016). Polyethylene glycol, Selexol®, and Genosorb® (mixtures of dimethyl ethers and polyethylene glycol) are the most commonly used solvents for biogas absorption, and this system also removed water, hydrogen sulphide, nitrogen, and halogenated hydrocarbons along with carbon dioxide before the purification process (López et al., 2012). The absorption process occurs at lower pressure (mainly 4–8 bars) and results in a lower energy compared to the water scrubbing (6–10 bars). The organic scrubbing technique is much less energy-consuming than the water scrubbers (Biernat and Samson-Bręk, 2011).

Regeneration of organic solvents is a complex process compared to water, because pressure release and air stripping are not effective for regenerating organic solvents (Allegue and Hinge, 2012). In practice, the organic solvent (solutions of polyethylene glycol) from the scrubbing medium is recirculated into the system and regenerated by heating to 40 and 80 °C, requiring an additional energy of 0.1–0.15 kWh/Nm^3^ of the biogas or/and the gradual reduction of pressure (Ryckebosch et al., 2011; Nguyen et al., 2020). Likewise, a large amount of energy is required to regenerate the organic solvent from hydrogen sulphide (López et al., 2012). This is because hydrogen sulphide is highly soluble in the organic solvent and during regeneration at a higher temperature (approximately 50 °C) is desired. In this system, methane concentrations in the product gas stream are in the range of 93–98% with >2% methane loss within the exhaust gas stream (contains hydrogen sulphide), due to environmental legislation, further cleaning steps are required for its removal (Allegue and Hinge, 2012). The easiest way to reduce hydrogen sulphide post-treatment difficulty is by applying pretreatment to the raw biogas prior to absorption (Hoyer et al., 2016). The advantage of this technique is that no additional drying process is required, because of the absorption of water by the organic solvent (Allegue and Hinge, 2012).

#### 5.2.3 Chemical scrubbing techniques

Chemical absorption (Figure 2C) is based on the principle of reversible chemical reactions between carbon dioxide and the chemical adsorbents present in the liquid-phase, which are alkanol amine solutions such as mono-ethanol amine (MEA) di-methyl ethanol amine (DMEA), diethanolamine (DEA), tertiary amines, and other amine compounds (Nguyen et al., 2020). These solvents can be used to dissolve carbon dioxide, which is not a simple dissolving process, but they react chemically with them and drive them into a solution (Kumar et al., 2002; López et al., 2012; Allegue and Hinge, 2012). The chemical adsorbents are selectively reacted with carbon dioxide so that the CH_4_ loss is insignificant (<0.1%) after it dissolves in the solvent solution (Sun et al., 2015; Nguyen et al., 2020) due to evaporation (Biernat and Samson-Bręk, 2011), thereby there is no requirement for post-combustion of the lean gas. Ideally, ammonia and hydrogen sulphide can also be removed, because the chemical solvents are toxic to the environment and affects public health. Several authors reported that using the chemical scrubber technique, it is possible to produce 99% by volume methane purity (Petersson and Wellinger 2009; López et al., 2012), while if there is no O_2_ or/and N_2_ in the biogas inflow, it can be increased to 99.5% (Allegue and Hinge, 2012).

Hydrogen sulphide produces a corrosive chemical reaction with amine solution that results in the degradation of amine. Its removal should therefore be recommended (Vega et al., 2014; Nguyen et al., 2020). Hydrogen sulphide can be oxidized into elemental sulfur using catalytic oxidation with chelated-iron salt solutions through the reduction of soluble ferric chelated iron (Fe^3+^) into ferrous iron (Fe^2+^) (see reaction below). In this way, the chelating agents (either Fe^3+^ or Fe^2+^) prevent the precipitation of iron hydroxide or iron sulphide when Fe^2+^ can be re-oxidized to Fe^3+^ simply by air stripping, where Fe^3+^ participating in the absorption process acts as a catalyst. In this system, about 99.99% of sulphide removal efficiencies can be achieved (Allegue and Hinge, 2012).

Purification: H_2_S+2[Fe^3+^] S + 2[Fe^2+^] + 2H^+^

Regeneration: 2[Fe^2+^] + 0.5O_2_ + 2H^+^2[Fe^3+^] + H_2_O

Carbon dioxide can also be absorbed using caustic soda (NaOH) mainly applied for the simultaneous removal of carbon dioxide and hydrogen sulphide, while the higher technical requirements to study with the caustic soda solution are hardily applied where large volumes of water can be contaminated with sodium sulphide that requires further disposal (Allegue and Hinge, 2012). Due to the environmental legislation with regard to the disposal of contaminated absorbers and absorber chemicals, the absorbers (amines) should be regenerated either using heat (steam) or vacuum (López et al., 2012). For regeneration of the carbon dioxide saturated amine solution, it should be heated to 120–160 °C in the desorption column and cooled down to 40 °C before the start of the new absorption cycle (Allegue and Hinge, 2012). During regeneration, 0.5 kWh/Nm_3_ of cleaned biogas or 15–30% of the generated energy from the biomethane is consumed (Leung et al., 2014; López et al., 2012). This technique has an advantage since amines are highly selective (react only with CO_2_ as 2RNH_2_ + CO_2_ = RNHCOO^− +^ RNH_3_^+^), thereby the loss of methane is usually low (López et al., 2012). In this system, solvent regeneration requires an energy intensive process by breaking the strong chemical reactions between the gas molecules (Kapdi et al., 2004; López et al., 2012). Therefore, it is advisable to remove hydrogen sulphide before the absorption processes in the amine scrubber using iron compounds (a process having higher hydrogen sulphide removal efficiency and low chemical consumption (Biernat and Samson-Bręk, 2011).

### 5.3 Membrane separation technology

Unlike the conventional membranes for methane and carbon dioxide separation (that are densely polymeric membranes built on the “solution-diffusion mechanism”), the membranes used in this study are materials made permeable to carbon dioxide, water, and ammonia (Deng and Hägg 2010). In this case, oxygen and hydrogen sulphide permeate to some extent through the membrane fiber, while nitrogen and methane pass only to a very low level of extent (López et al., 2012). Membrane based biogas separation follows the fact that gases have different permeabilities.

**Figure 3:**
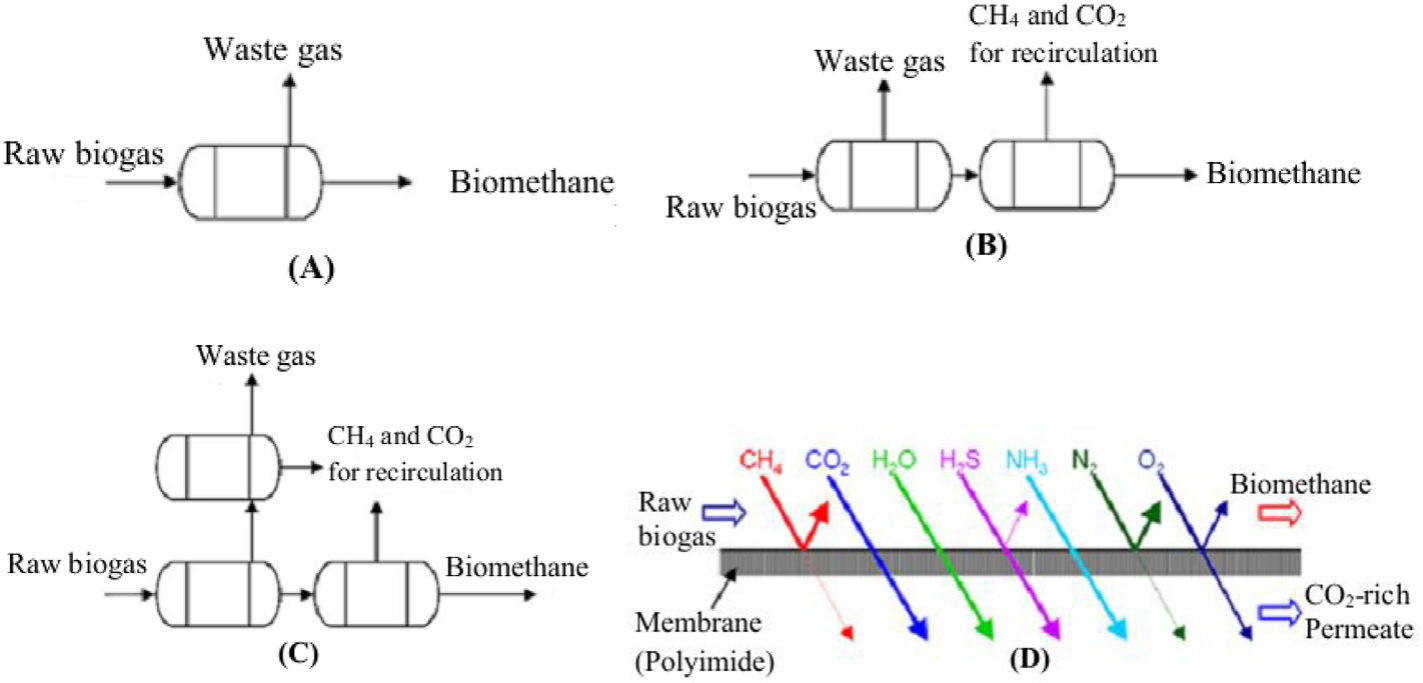
Different process schematics of membrane configurations in a biogas upgrading system (A–C): (A) one stage, (B) two stage, (C) three stages cascades and (D) principles of membrane separation technology (adapted from Allegue and Hinge, 2012; Hoyer et al., 2016).

Methane and carbon dioxide gas molecules travel through the membrane at different rates, and carbon dioxide has a higher permeability than methane (which can separate these gas mixtures accordingly) (Hoyer et al., 2016). The membrane separation is based on the principle that gases permeate through the membrane pores at different selectivities, where the membrane is impermeable to methane (large molecule) and permeable to carbon dioxide (small molecules). The difference in partial pressure between various gases found in the biogas is the driving force behind the separation process (López et al., 2012), where the difference in particle size or affinity of a certain molecules can be transported through the membrane, while other gases can’t (Figure 3D) (Hullu et al., 2008). Membrane separation occurs in a variety of designs, with typical operating pressures ranging from 7 to 20 bar (Peppers et al., 2019), resulting in higher pressure in the produced biomethane (Hoyer et al., 2016). As a general overview, the membrane suitability is 20 times permeable to carbon dioxide than to methane. From membrane separation, the exhaust gas rich in carbon dioxide can be produced with high purity with 99.9% carbon dioxide being achieved (which can be used in beverage and food industries) (Esposito et al., 2019); after cooling to -30 °C, oxygen, nitrogen, and trace methane are being separated (Nguyen et al., 2020). The membrane technique allows the separation of pollutants, mainly, carbon dioxide as well as hydrogen sulphide, water, and ammonia that are transported through a thin layer membrane, and methane is retained owing to the difference in affinity or/and particle size (Allegue and Hinge, 2012). This technique is still an emerging technology, having limited practical experience in biogas upgrading (Biernat and Samson-Bręk, 2011).

If the tube bundles are linked in 2-stage or 3-stage cascades (Figure 3(A–C)), the membrane can be maintained at higher methane purity and provide higher recovery potential than the single cascades. Hence, in the 2-stage system, the circulation loop returns the gas from the first membrane to the inlet and the “enriched methane” continues to the next membrane (Nguyen et al., 2020). In some laboratory studies (Makaruk et al., 2010; Baena-Moreno et al., 2020), with energy requirements between 0.18-0.33 kWh/Nm^3^ of biogas, the methane loss was up to 2%. Peppers et al. (2019) recently demonstrated the feasibility of membrane separation for a 100 Nm^3^/h biogas plant and recommend that pre-treatment of other gases is required to maintain the safety of the membrane and ensure higher purity of biomethane (Peppers et al., 2019; Baena-Moreno et al., 2020; Nguyen et al., 2020). The properties of these separation techniques strongly depend on the type of membranes used (López et al., 2012) with their particular specifications (Allegue and Hinge, 2012). Thus, the membrane separation techniques are classified under two categories: 1) high pressure (gas–gas) separation, where the membrane has a gas phase on both sides, and 2) gas-liquid absorption separation, with the liquid absorbing the diffused molecules (Allegue and Hinge, 2012).

#### 5.3.1 Gas–gas separation (dry or solid) membrane processes

For biogas upgrading, the dry membranes are made of materials that are permeable to carbon dioxide, water, oxygen, and ammonia. They permeate to some extent and to a low extent to methane and nitrogen through the membrane. Before the biogas enters the hollow fiber membrane, it first passes through the filter (mainly activated carbon) that was used to retain the oil droplets, water, aerosols, and hydrocarbons. Otherwise, these pollutants may affect the performance of the membrane (Biernat and Samson-Bręk, 2011). However, the imperfect separation of raw biogas using this technique results in a low biomethane yield of 92% in one step (Allegue and Hinge, 2012), whereas a continuously operating system in three stages (Figure 3(iii)), with selective separation of carbon dioxide, methane, and hydrogen sulphide, increases the methane yield to more than 96% (López et al., 2012). Besides, the purity of the upgraded methane gas can be improved by adding its size or by increasing the number of membrane modules, whereby a large amount of methane will permeate through the membrane and be found as methane lost. These losses can be mitigated in part by recirculating a portion of the permeated carbon dioxide-enriched gas. However, when numerous membrane modules are linked in series, the best result is achieved with recirculation of the permeated gas only from the last module (Allegue and Hinge, 2012).

#### 5.3.2 Gas–liquid absorption membranes

The gaseous from the liquid phase can be separated using micro-porous hydrophobic membrane separators, where low-pressure “gas–liquid membrane” processes are an auspicious technology for the removal of hydrogen sulphide (Allegue and Hinge, 2012). The molecules from the biogas stream flowing in one direction and able to diffuse through the membrane fiber are absorbed on the opposite side by liquid flowing in the counter current. However, due to the slight pressurization of the gas, the liquid is prevented from flowing to the gas side at an atmospheric pressure of 100 kPa which permits very high selectivity. If an amine solution is used in this technique, the biogas with 55% methane can be upgraded to more than 96% in one stage (Allegue and Hinge, 2012). To increase the membrane life time, hydrogen sulphide should be removed before high pressure (gas-liquid) membranes by cleaning with activated carbon before the membrane separation (Petersson and Wellinger 2009). However, due to their high cost, membranes are not yet competitive for the selective removal of hydrogen sulphide together with carbon dioxide (Allegue and Hinge, 2012). The hydrogen sulphide free (that is removed in the pretreatment) biogas is subjected to further purification using gas permeable membranes. In this technique, carbon dioxide will be permeated through the membrane at a faster rate than the other separation membranes (Petersson and Wellinger 2009).

### 5.4 Cryogenic separation technology

Cryogenic treatment is based on the difference in sublimation and boiling points between the biogas impurities (specifically, carbon dioxide and methane) (López et al., 2012; Prussi et al., 2019; Paglini et al., 2022). This technique is employed where gases could become liquid (condensed) or solid (re-sublimate) at higher pressures and lower temperatures, where methane and carbon dioxide have different condensation temperatures (Tan et al., 2017). Cryogenic separation is an emerging technology (López et al., 2012) and its principle is implemented in 4 phases: drying, compression, gas cleaning, and carbon dioxide removal. First, the incoming biogas is compressed to 17–26 bar pressure, consequently cooled to −25 °C (Allegue and Hinge, 2012). In this phase, hydrogen sulphide, water, sulfur dioxide, siloxanes, halogens, and other undesirable components are removed from the raw biogas. Then, the gas is further subjected to coalescence filtering. Upon catalyst addition, the remaining contaminants are removed. Carbon dioxide is removed in two stages: first, the biogas is cooled to lower temperatures (−50 and -59 °C), and up to 30–40% of the carbon dioxide is removed as liquid. In a subsequent phase, the retaining gas stream is cooled to −85 °C to solidify carbon dioxide (Allegue and Hinge, 2012), allowing carbon dioxide to be separated from the biogas in solid or liquid form, while methane accumulates in the gas phase and the separated carbon dioxide is clean and used/sold for further applications (Biernat and Samson-Bręk, 2011).

**Figure 4:**
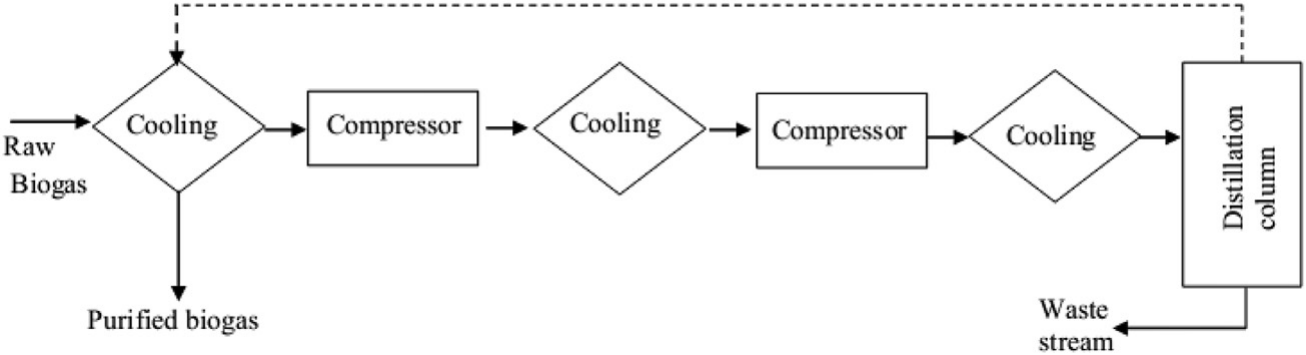
Schematics of cryogenic separation techniques (adapted from Biernat and Samson-Bręk, 2011).

This system is technically very demanding, but it can produce very pure methane and carbon dioxide (for both up to 99.9% by volume) within <1 % methane loss and 0–5% electrical energy demand (Nguyen et al., 2020). This technique is an environmentally friendly technique, because no chemicals are used. Before starting the cryogenic upgrading process, it is recommended to remove hydrogen sulphide and water vapor from raw biogas in the pretreatment step. Otherwise, it causes freezing and may damage the heat exchangers (Hoyer et al., 2016) by clogging of the system (Allegue and Hinge, 2012). While siloxanes and volatile organic compounds (VOC) are efficiently removed during the cooling and condensation process (i.e. natural part of the cryogenic biogas upgrading process), it is also used to remove trace contaminants such as oxygen and nitrogen) from the landfill gas (Hoyer et al., 2016). Figure 4 illustrates the schematics of cryogenic separation techniques applied in the biogas upgrading process. The advantage of cryogenic treatment is the possibility to produce biogas with a high CH_4_ content of up to 99%, while it also uses a lot of technological equipment (i.e. compressors, heat exchangers, and turbines) and the substantial demand for equipment makes this treatment extremely expensive (Biernat and Samson-Bręk, 2011). However, the disadvantage of this technique is that higher energy is needed (mainly up to 10% of methane produced) for compression and refrigeration of the raw biogas, while if the produced biomethane is going to be liquefied (at −125 °C and 15 bar), the energy required to condense the initial biogas can be recovered (Nguyen et al., 2020). As well, the solid frozen carbon dioxide can be utilized as dry ice for further industrial applications (Esposito et al., 2019).

### 5.5 Emerging biological technologies

#### 5.5.1 Biocatalysis techniques

As described by Nguyen et al. (2020), this technique is promising in capturing carbon dioxide, but has no application yet for biogas upgrading into biomethane. Biocatalysis is a common technique mainly in removing carbon dioxide using common enzymes called “carbonic anhydrases”, to convert water and carbon dioxide (from the raw biogas) into bicarbonates, which thereby reduces the cost of energy supply during the removal step (Nguyen et al., 2020). The energy requirement in the chemical absorption technique is determined via the capability of the solvents (in taking up carbon dioxide and its specific heat of reaction). Higher energy savings could be achieved when a solvent has a higher capacity and lower heat of reaction (Gundersen et al., 2014; Kunze et al., 2015). For example, alkali carbonates and amines are the potential solvents, but suffer from unhurried absorption kinetics (Kunze et al., 2015), while enzymes are activators or catalysts used in enhancing the absorption kinetics of carbon dioxide. In such a case, the formation of bicarbonates during the conversion is a rate limiting step of carbon dioxide absorption (Beiron et al., 2019). The absorption capacity (absorbed volume) has been improved by a factor of >4 by the addition of carbonic anhydrase at 0.2–30 wt. % with K_2_CO_3_ and MEA to the raw biogas (Kunze et al., 2015), and increased by a factor of 3 for MDEA absorption by the addition of catalytic enzyme (carbonic anhydrase) (Vinoba et al., 2013).

#### 5.5.2 Microalgae-based photobioreactors

The microalgae (as autotrophic microorganisms) have the capability to fix carbon dioxide in the presence of sunlight and, upon nutrient utilization, produce new biomass (Xu et al., 2017). An algal-bacterial photobioreactor is the most recent development that can be carried out for the simultaneous removal of carbon dioxide and other trace components like hydrogen sulphide (Hoyer et al., 2016). Because microalgae have a higher capacity for carbon dioxide fixation, biological carbon dioxide sequestration in biogas upgrading using photosynthetic microalgae (in the photobioreactor) has received a lot of attention (Xu et al., 2017). The microalgae-based technique is an emerging technology developed in recent years, and applied for simultaneous wastewater treatment and biogas upgrading. Because of its high economic and environmental convenience, this technique is advantageous (Xu et al., 2017). A limited number of studies have been conducted on using microalgae cultures in an integrated anaerobic digestate wastewater treatment followed by biogas upgrading (Converti et al., 2009; Xu et al., 2017; Nagarajan et al., 2019; Bose et al., 2020).

**Figure 5:**
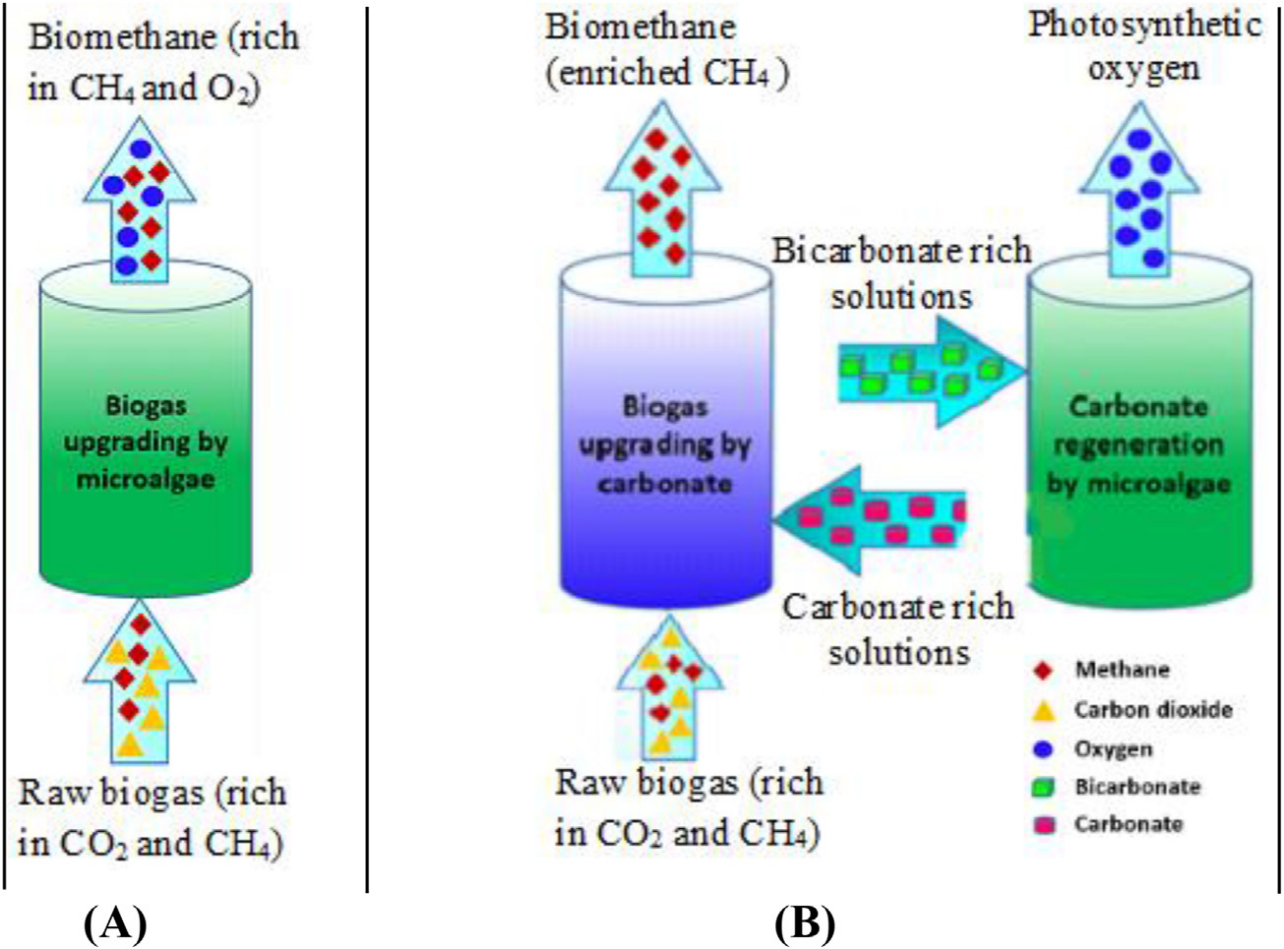
Schematics of microalgae-based photobioreactor in different approaches: (A) direct biogas upgrading using microalgae, and (B) indirect biogas upgrading using microalgae with carbonate/bicarbonate cycle approaches (adapted from Nguyen et al., 2020).

In the integrated systems, the anaerobic digester that produces raw biogas is fed into the photobioreactor, where the carbon dioxide is taken up by the microalgae in a direct approach (Figure 5A), and thereby the crude biogas is upgraded in an economically feasible process when applied in combination with wastewater treatment (aiming at biogas generation) (Xu et al., 2017). Converti et al. (2009) first introduced this configuration through the linkage of an anaerobic digester of a mixed sludge followed by a photobioreactor that leads to the production of biogas with a methane content of above 70%. Various authors achieved a higher content of methane in the upgraded biogas using a similar setting of an integrated (wastewater treatment-biogas upgrading scheme) (Yan and Zheng, 2013; Xu et al., 2017; Nagarajan et al., 2019; Bose et al., 2020).

The C/N ratio is very significant for the growth of microalgae once the co-cultivation with bacteria or fungi has been carried out (Xu et al., 2017). In addition, at higher C/N ratios of the influents, this technology achieves low removal efficiency of phosphate and nitrogen in the wastewater, and thereby the microalgae growth rate is limited. Besides, the microalgae growth is also affected by the nutrients and organic matter concentrations and the occurrence of heterotrophic microorganisms in the wastewater. The algal-based photobioreactor is advantageous in terms of cost-effectiveness and environmental friendliness when compared to conventional biogas enhancing and sewage treatment techniques, though some limitations exist, such as methane loss, challenges in harvesting microalgae biomass, energy consumption, and/or leading to an increase in effluents (Xu et al., 2017). As it was explored by Nagarajan et al. (2019) and Bose et al. (2020), the limitation of the direct method (Figure 5A) could be alleviated by applying an indirect biogas upgrading system (Figure 5B), where carbon dioxide can be taken in a carbonate solution, whereas its saturated solution is fed into microalgae culture. The bicarbonate has been utilized by the microalgae (as a carbon source for growth), and the carbonate is being regenerated for the subsequent biogas upgrading cycle (Xia et al., 2015). However, the principle of this approach has been restricted only to a limited number of specific microalgal species (that tolerate the alkali environment and higher ionic strength (Xia et al., 2015; Nguyen et al., 2020).

Xu et al. (2017) applied three different treatment techniques, namely algal-bacterial, mono-algae, and algal-fungal cultures to synthetic domestic sewage for the removal of carbon dioxide from biogas in a photobioreactor system. The highest average methane content of 93.25 ± 3.84% (v/v) was achieved in the upgraded biogas when using algal-fungal culture. Besides, at an influent C/N ratio of 5:1, the algal-fungal cultures achieved an average 80.23 ± 3.92%, 78.41 ± 3.98% and 75.85 ± 6.61% of COD, total phosphorus and total nitrogen removal efficiency, respectively; while the algal-bacterial culture also achieved 82.28% total nitrogen removal efficiency. These findings will confirm that the microalgae-based photobioreactor is a reference for simultaneous wastewater treatment and biogas upgrading (Xu et al., 2017). By applying an optimization of the cultural conditions using *Chlorella* sp. microalgae attained a methane content of 92% (Yan and Zheng, 2013). Rodero et al. (2019) also investigated the performance of an algal-bacterial photobioreactor in a semi-industrial scale treatment of centrate wastewater type at a maximum liquid to gas (L/G) ratio of 3.5 and achieved the highest removal efficiencies of 99% and 100% for carbon dioxide and hydrogen sulphide, respectively, where the maximum upgraded biomethane content was 90% (the limitation was due to oxygen and nitrogen desorption).

In another study by Mann et al. (2009), the biogas conditioning has been carried out at a laboratory-scale photobioreactor using the microalgae *Chlorella sp.* and found up to 97.07% carbon dioxide and hydrogen sulphide removal efficiency, respectively. Toledo-Cervantes et al. (2022) carried out a continuous study to evaluate the performance of a tabular photobioreactor linked with a gas absorption column for the reduction of carbon dioxide from biogas. The results showed that up to 98% and 99% of carbon dioxide and hydrogen sulphide removal efficiency was achieved in an alkaline environment (pH ∼ 10).

## 6 Removal of other biogas impurities

**Figure 6:**
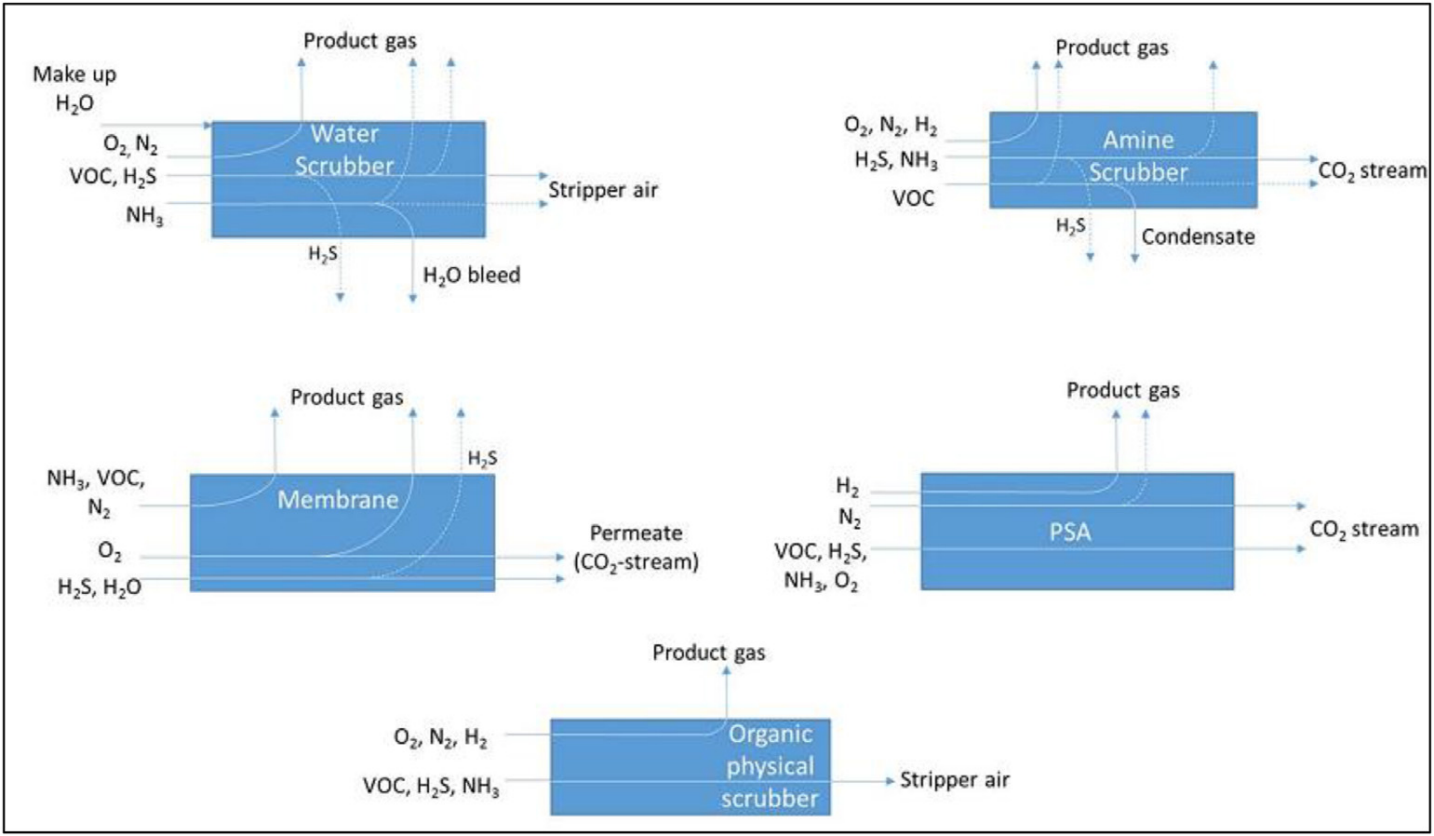
The separation pathways of various biogas impurities through different biogas upgrading technologies (Hoyer et al., 2016).

Apart from carbon dioxide and methane, raw biogas involves undesirable contaminants, which are referred to as biogas impurities, including hydrogen sulphide, water vapor, nitrogen, oxygen, siloxanes, ammonia, and particulates (Allegue and Hinge, 2012). To use biogas as a vehicle fuel by enhancing its energy value (to provide longer driving distances using fixed volume gas storage), it has to be enriched with methane, achieved primarily by removing carbon dioxide (Hoyer et al., 2016). These impurities may cause various undesirable effects in the upgrading process and have various health problems (Macor and Benato, 2020a; 2020b) and must be removed depending on whether they are unglued together with carbon dioxide during the biogas upgrading or during pretreatment (i.e., before the carbon dioxide removal begins), or in the upgraded biomethane (Hoyer et al., 2016), or depending on the existing regulations. Figure 6 shows an indicative overview of the separation pathways of different biogas impurities over different upgrading techniques.

### 6.1 Removal of water

Water can be removed from biogas using various techniques such as cooling, compression, and adsorption and absorption methods. By refrigerating through heat exchangers, usually by increasing pressure and at decreased temperature (Biernat and Samson-Bręk, 2011). Thereby, the condensed water droplets are removed as wastewater based on the existing environmental standards or recycled into the digester (Ryckebosch et al., 2011; Allegue and Hinge, 2012). The biogas moisture can also be removed by using adsorption dryers using high water dryer adsorbents, for example, silica gel, aluminum oxide, activated carbon, magnesium oxide, and molecular sieves, whereas adsorbent regeneration can be done through heating (Nguyen et al., 2020). Amongst them, adsorption via zeolites/molecular sieves, or alumina is the most common chemical drying method (Allegue and Hinge, 2012). Water absorption is also another common technique, which can be applied using water binding constituents like glycol or triethylene glycol as well as hygroscopic salts. These salts are then dissolved by absorbing moisture from biogas, and the saturated salt solution is released from the bottom side of the vessel (Allegue and Hinge, 2012). Water-soluble aerosols and gaseous impurities are removed from biogas at the same time as water (Petersson and Wellinger, 2009a, Petersson and Wellinger, 2009b; Biernat and Samson-Bręk, 2011; López et al., 2012).

### 6.2 Removal of hydrogen sulphide

Hydrogen sulphide is the main biogas impurity produced during the reduction of sulfur compounds (amino acids, peptides, sulfates) through microbial action. Hydrogen sulphide removal is carried out through the range of treatment steps via primary to tertiary steps (Hoyer et al., 2016). This method is mainly used in digesters having higher concentrations of sulphur whereby the amount of hydrogen sulphide in the biogas is expected to be higher (>1000 mg/L) (Petersson and Wellinger, 2009a, Petersson and Wellinger, 2009b). The primary step is the precipitation of hydrogen sulphide using the addition of iron salts, Fe^2+^ or Fe^3+^ ions in the form of [FeCl_3_, FeSO_4_ or FeCl_2_] into the digester, which produces an insoluble iron sulphide precipitate (FeS) to be removed simultaneously with the digester (Petersson and Wellinger, 2009a, Petersson and Wellinger, 2009b) and prevents its transport further into the downstream. Accordingly, the hydrogen sulphide removal in the primary step can be made to reach hydrogen sulphide concentrations of 50–200 ppmv (Hoyer et al., 2016). The secondary and tertiary steps are the biological and activated carbon adsorption techniques, which have been applied to remove hydrogen sulphide from biogas.

The biological processes are carried out by inoculating a strain of bacteria (for example, *Thiobacillus* and *Sulfolobus*) that feeds the sulphur on a carrier material (i.e. filling plates or bark). This technique is mostly used when there is only a small amount of hydrogen sulphide emissions (Hoyer et al., 2016), whereas the most common technique is tertiary removal, which is based on the interaction of the pore surface of activated carbon and hydrogen sulphide in the presence of oxygen to produce hydrogen sulphide (Hoyer et al., 2016). This process is carried out in the gas phase at lower pressures and temperatures, while water condensation (in the biogas and activated carbon) is controlled by heating before the process of adsorption is carried out; whereas both chemical and physical adsorption have occurred in the activated carbon filters (Hoyer et al., 2016).

### 6.3 Removal of siloxanes and particulates

Siloxanes are water-insoluble and have a remarkable high vapor pressure (Paolini et al., 2018a). Volatile siloxane like hexamethyldisiloxane is present to some extent in the biogas (mainly in the sewage sludge up to 50 mg/Nm^3^ (beyond the limits set by engines of 15 mg/Nm^3^) and in the landfill gas (Hoyer et al., 2016). When siloxane is in contact with the dryers, it adsorbs on the adsorbent (like zeolite) pore surface (in the dryer) and is used as a desiccant. During regeneration of the dryer, siloxanes are subjected to decomposition via heating and found to silicone precipitate (which leads to fouling through pore surface blockages in the long term and then lowers the effective rate of desiccant (Hoyer et al., 2016). The traditional methods of siloxane removal from biogas are either by water scrubbing (mainly for the volatile hexamethyldisiloxane) or using hot sulfuric acid through the scrubbing process (Schweigkofler and Niessner, 2001; Hoyer et al., 2016). An appropriate method for the high level of siloxane is primarily by chilling the biogas to −30 °C and thereby via adsorption using molecular sieve or activated carbon such as silica gel (Hoyer et al., 2016). When the biogas is quite free of VOC, the removal of siloxane is performed using the vacuum swing adsorption process, while temperature swing adsorption might be used if both of them exist together in the biogas, and the vacuum is very useful in the adsorption bed regeneration (Inc, 2014).

A study conducted by Schweigkofler and Niessner (2001) achieved approximately 95% removal efficiency of siloxanes by chemical absorption techniques using a mixture of 650 L/m^3^ nitric acid and 480 L/m^3^ sulfuric acid at 60 °C. Besides, about 98% of siloxanes can be removed using selexol from a biogas plant (Wheless and Pierce, 2004). In another study, cryogenic separation technology had been effectively removed siloxanes at different temperatures, where the highest and lowest removal effeicncy had been achieved at −70 °C, and 5 °C, respectively for 99.3% and 12% (Ruiling et al., 2017). As reported by Schweigkofler and Niessner (2001) a good adsorption capacity for siloxanes removal have been achieved using different adsorbents such as silica gel, molecular sieves, polymer beads and activated carbon. Membrane separation techniques are also commonly achieved above 80% removal of siloxanes (Favre et al., 2009). Piechota (2021a) studied a cryogenic temperature-condensation system ranged from +40 to 50 °C at various flow rates of biogas achieved 99.87% removal efficiency of siloxanes. The same author had been performed the removal of siolxanes completely and achieved 99.76% removal of non-siloxanes impurities using adsorptive packed column system from raw biogas (Piechota (2021b).

Particulates such as oil and dust exist from the compressors, and are removed through filtration at 2–5 μm. For the removal of particulates, a proven filtration technology has been used, such as passing the biogas through a filter pad made of wide stainless steel, using a suitable cyclone separator, or using packs of ceramic filters (Allegue and Hinge, 2012).

### 6.4 Removal of ammonia, nitrogen and oxygen

Ammonia is formed as an end-product of biogas during the biodegradation of proteins, and its amount depends on pH and the composition of the substrates in the digester (Hoyer et al., 2016). At lower ammonia concentrations, a separate removal step is not required, as it is simultaneously removed by gas drying in the adsorption process of the biogas upgrading schemes. Due to the higher ammonia solubility in water, ammonia is removed together with water, while a complete removal can be successfully achieved without the requirement of a pretreatment step, when scrubbers are used as biogas upgrading techniques (Allegue and Hinge, 2012; Nguyen et al., 2020). A horizontal biotrickling filter filled with the activated carbon inoculated with nitrifying bacteria to oxidized sulfur had been employed for the co-removal of biogas pollutants achieved 95% H_2_S and 98% NH_3_ at an operating range of 20–100 ppm and gas resident time of 8 and 4 s, respectively (Jiang et al., 2009). Besides, the water scrubbing can be effectively removed sulfur, halogens and ammonia, while PSA co-removed water vapour and ammonia from raw biogas (Ullah et al., 2017).

In the biogas digester, oxygen is not simply present because oxygen could be consumed through the facultative aerobic microbial, while the landfill gas constitutes nitrogen and oxygen when the landfill gas has collected under pressure (Petersson and Wellinger, 2009a, Petersson and Wellinger, 2009b). As explained by Petersson and Wellinger, 2009a, Petersson and Wellinger, 2009b the adsorption technique can be removed both N_2_ and O_2_ from biogas using activated carbon and molecular sieve, the membrane separation technique and PSA that are designed to remove CO_2_ and H_2_S could remove some fractions of N_2_ and O_2_. These gases can be removed using adsorption via activated carbon, membrane separation, and molecular sieves, while the desulphurisation process also removes them to some extent (Nguyen et al., 2020).

### 6.5 Removal of halogenated compounds and hydrocarbons

The removal techniques of halogenated hydrocarbons and VOCs are in a similar way to those applied to the removal of carbon dioxide in biogas upgrading technologies (Hoyer et al., 2016). A special technology applied for halogen removal is the adsorption method by passing the biogas through “pressurized tube exchangers” filled with specific adsorbent materials like activated carbon. Hence, small molecules like carbon dioxide and methane will pass through the column, while big molecules (halogens) will be adsorbed in the first vessel (treating the biogas), while the second vessel is used for desorbing (regeneration) of the pollutants by heating to 200 °C (Wellinger and Lindberg, 2000; López et al., 2012). Then, the adsorbed impurities are evaporated through the flow of inert gas, which requires further treatment due to the existing environmental standards (Allegue and Hinge, 2012).

## 7 Conclusions and future perspectives

Unlike coal and fossil fuels, biogas is among the renewable energy resources that are growing rapidly as a promising, economically feasible and environmentally benign alternative made through the anaerobic digestion of organic water and sludge. In developing countries, biogas technology has been developed promptly to substitute the drawbacks of wood based solid fuels in terms of health and environmental implications. However, biogas contains various impurities, including carbon dioxide (main constituent of biogas), and small fractions of other gas contaminants (like nitrogen, carbon monoxide, hydrogen sulphide, oxygen, ammonia, and trace contents of siloxanes and halogenated hydrocarbons). Depending on the emission level, these impurities result in various direct/indirect environmental impacts. To tackle these problems, different commercially available and emerging technologies are being developed worldwide to remove carbon dioxide (via biogas upgrading) and other biogas impurities to enrich the methane content. The use of biomethane as a product of upgraded biogas (by removing carbon dioxide and other constituents) has emerged as an alternative mitigation strategy to reduce the problems of fossil fuel-based energy demand.

The commercially available technologies applied in removing biogas impurities are pressure swing adsorption, membrane separation, water scrubbers, chemical scrubbers, physical organic solvent scrubbers, cryogenic separation, and other emerging biotechnological platforms (like photobioreactor and biocatalysis). Amongst them, physical organic scrubbers and membrane-based separation perform the simultaneous removal of carbon dioxide with other impurities (CO_2_/CH_4_ and H_2_S/CH_4_), while the pressure swing adsorption and amine-based chemical absorption require a pretreatment stage, and others, like cryogenic and membrane separations, require post-treatment after the biomethane has been produced. The water scrubbing technique can reduce the level of H_2_S from biogas, but it has a limitation to remove the organic sulfur fractions by forming a solid sulfur deposits. The membrane and cryogenic separation provides higher biomethane purity standards and are interesting for large-scale plants, but. Micro-algae based photobioreactor system is a promising alternative for its low cost and co removals (CO_2_ and H_2_S), but not yet tested at large-scale applications in separating the impurities from biogas. Further research will be required to adopt: i) low-cost, environmentally friendly, and long-term technologies for integrating pre- and post-treatment with biogas upgrading technologies, ii) hybrid systems, by integrating a promising and emerging technologies (like photobioreactor or membrane separation with other processes) to achieve higher removal of impurities and to enhance the recovery potential of biomethane compared to single step processes.

## Declarations

### Author contribution statement

All authors listed have significantly contributed to the development and the writing of this article.

### Funding statement

This work was supported by Mekelle University (Ethiopia) under grant agreement number of CRPO/MU-CHS/Rec/Medium/3/2019–2021.

### Data availability statement

Data will be made available on request.

### Declaration of interest’s statement

The authors declare no conflict of interest.

### Additional information

No additional information is available for this paper.
